# Protein Structure and Evolution: Are They Constrained Globally by a Principle Derived from Information Theory?

**DOI:** 10.1371/journal.pone.0125663

**Published:** 2015-05-13

**Authors:** Leslie Hatton, Gregory Warr

**Affiliations:** 1 Faculty of Science, Engineering and Computing, Kingston University, London, UK; 2 Medical University of South Carolina, Charleston, South Carolina, USA; University of Edinburgh, UNITED KINGDOM

## Abstract

That the physicochemical properties of amino acids constrain the structure, function and evolution of proteins is not in doubt. However, principles derived from information theory may also set bounds on the structure (and thus also the evolution) of proteins. Here we analyze the global properties of the full set of proteins in release 13-11 of the SwissProt database, showing by experimental test of predictions from information theory that their collective structure exhibits properties that are consistent with their being guided by a conservation principle. This principle (Conservation of Information) defines the global properties of systems composed of discrete components each of which is in turn assembled from discrete smaller pieces. In the system of proteins, each protein is a component, and each protein is assembled from amino acids. Central to this principle is the inter-relationship of the unique amino acid count and total length of a protein and its implications for both average protein length and occurrence of proteins with specific unique amino acid counts. The unique amino acid count is simply the number of distinct amino acids (including those that are post-translationally modified) that occur in a protein, and is independent of the number of times that the particular amino acid occurs in the sequence. Conservation of Information does not operate at the local level (it is independent of the physicochemical properties of the amino acids) where the influences of natural selection are manifest in the variety of protein structure and function that is well understood. Rather, this analysis implies that Conservation of Information would define the global bounds within which the whole system of proteins is constrained; thus it appears to be acting to constrain evolution at a level different from natural selection, a conclusion that appears counter-intuitive but is supported by the studies described herein.

## Introduction

Systems composed of discrete pieces, often arranged in a sequence, are ubiquitous and in origin are both natural and man-made. Examples from nature include the systems of nucleic acids, proteins and complex carbohydrates. The proteins, while themselves discrete components of a system, are individually assembled from smaller discrete pieces i.e. the amino acids. Historically, the biological polymers of proteins, DNA and complex carbohydrates have been studied primarily in the reductionist and mechanistic context of biochemistry and genetics, thereby producing ever-increasing and finer-grained insights into their properties.

However, such systems can also be analyzed from a global perspective, using concepts from information theory [1–6] and statistical physics [7–9] that demonstrate amongst other things the extraordinary ubiquity of power-law distributions in biological and many other systems [10]. Indeed Karev et. al [1] specifically note that “The question that emerges when the same mathematical structure appears in apparently unrelated contexts is: are these formal similarities coincidental and superficial or do they reflect a deep connection at the level of evolutionary mechanisms?” We will argue here that in the case of proteins such a connection exists; however, the evidence suggests that the connection is at a global level and results from the action of a conservation principle (Conservation of Information) derived from information theory. The counter-intuitive implication is that the Conservation of Information sets boundaries within which the evolutionary mechanisms operate.

Conservation principles and their associated symmetries have long been known to be fundamental in the evolution of physical systems [11] and the relationship between local effects and the global constraint of conservation principles is well understood in for example the operation of the general gas equation, in which the pressure, volume and temperature are constrained through a universal constant, independently of the local motion of the molecules. In a biological system, the operation of the laws of thermodynamics on biochemical and biophysical systems is well understood, in which local violations (e.g. the reversal of entropy characteristic in life forms) are permitted but in which the global system nevertheless adjusts so that the second law of thermodynamics is not violated.

In this study we address whether the global structure of a system of proteins is constrained by the Conservation of Information, which was recently shown to operate in systems of computer software [12]. We were led to ask this particular question of proteins because the Conservation of Information applies to systems that possess a feature that is shared by proteins and computer software, i.e. the element of sequential choice. Both systems consist of components that are themselves assembled sequentially from parts, i.e. the amino acids in proteins, and tokens in software languages. This conservation principle was derived by merging Hartley-Shannon information and statistical mechanics [13–16]. It will be described more fully below, but it is important to note that the operation of the Conservation of Information is independent asymptotically of scale and of the meaning of the parts from which components are assembled. The irrelevance of token (here amino acid) meaning is an important part of Hartley-Shannon theory, indeed Hartley specifically defined *information* as “the successive selection of signs, rejecting all meaning as a mere subjective factor” [13].

In the context of proteins, this latter point eliminates from the analysis any consideration of the different physicochemical properties of the amino acids. This irrelevance of the physicochemical properties of the amino acid side chains is highly counter-intuitive, since they are the foundation of our understanding of protein structure and function. Nevertheless, we will here present evidence that Conservation of Information guides the global properties of a system of proteins regardless of the disparate physicochemical properties of individual amino acids and of the domain of life in which the proteins have evolved.

### The unique amino acid count and the Conservation of Information

In the Conservation of Information two parameters emerge by which global properties are categorized [12]. The first is both intuitively obvious and easily accessible, and for proteins is simply *t*_*i*_, the length in amino acids of the *i* ^*th*^ protein. The second parameter is less intuitive from a biological viewpoint and is consequently a little more difficult to explain and also to extract from protein databases. This is *a*_*i*_, a count of the *unique* amino acids used in building the *i* ^*th*^ protein, which will be defined below. In this notation, the information content of the *i* ^*th*^ protein is $log{\left(a_{i}\right)}^{t_{i}}=t_{i}loga_{i}$, or the log of the total number of possible orderings and considering amino acid properties as irrelevant.

The concept of the unique amino acid count of a protein is illustrated in Table 1 using sequences contrived for this purpose, and can be seen to be wholly independent of the physicochemical properties of the amino acids. The unique count is simply the number of distinct amino acids including those that are post-translationally modified (PTM) that occur in the sequence of a protein, in other words the protein’s alphabet. Repetitions of an amino acid in the same sequence are not counted and are marked as italicized in Table 1 because they appeared earlier in the sequence. The modification of amino acids by glycosylation merits specific mention here because the chemical structures of the glycosyl moieties that can be added as PTM to amino acids are both numerous and diverse. Each family of glycan, e.g. “Asparagine N-linked high mannose” contains a large number of structural variants [17] (www.unicarbkb.org). Thus, each distinct carbohydrate structure within the family would contribute 1 to the unique amino acid count of a protein in which it was present.

**Table 1:** The unique amino acid count defined for seven peptide sequences, including PTM amino acids. Italicised amino acids are repetitions in the same sequence. Abbreviations: Ala, Alanine; Ser, Serine; Tyr, Tyrosine; Ile, Isoleucine; pSer, phosphoserine; pTyr, phosphotyrosine; (Ser-O-GlcNAc), N-Acetylglucosamine-O-linked Serine

Unique amino acid count	Length	Sequence
3	3	Ala-Ser-Tyr
3	6	Ala-Ser-Tyr-*Ala*-*Ser*-*Tyr*
4	7	Ala-Ser-Ile-Tyr-*Ala*-*Ser*-*Tyr*
4	9	Ala-pSer-Tyr-*Ala*-Ser-*Tyr*-*Ala*-*Ser*-*Tyr*
5	6	Ala-pSer-Tyr-*Ala*-Ser-pTyr
5	6	Ala-pSer-Tyr-*Ala*-(Ser-O-GlcNAc)-pTyr
6	7	Ala-pSer-Tyr-*Ala*-(Ser-O-GlcNAc)-pTyr-Ser

To illustrate the concept of the unique amino acid count with sequences taken from nature, two small proteins, one from a virus and the other from a mollusc [18] are shown in Table 2 along with their sequences in their single letter abbreviations for compactness. Biologically, these two proteins are very different. They have different lengths, (23 and 45 amino acids respectively); they are built using different amino acids, (they have only 3 amino acids in common, alinine (A), lysine (K) and valine (V)); they have very distinct structures and functions. However, from the perspective of information theory, they share a fundamental organizing property: *each is composed of exactly 6 unique amino acids*. These are AEIKNV and AKPRSV respectively, and this number is the unique amino acid count of the protein. It does not matter if an amino acid is present once or more often in the sequence. If it is present at all, then it contributes a count of 1 to the unique amino acid count. This property is obviously independent of any physicochemical properties of the amino acids since the ordering of amino acids or number of any particular amino acid (provided there is at least 1 of that amino acid) is not considered in the Conservation of Information.

**Table 2:** Sequence of two small proteins.

Protein	Sequence
VG22_BPT2 (from Phage T2)	KAEEEVEKNK EEAEEEAEKK IAE
PHI_MYTCA (from the California mussel)	AKAKRSPRKK KAAVKKSSKS
	KAKKPKSPKK KKAAKKPAKK AAKKK

Clearly the number of possible choices is fundamentally important to information theory and the unique amino acid count of a protein must therefore be drawn not only from the 20 canonical amino acids encoded in the DNA plus the additional two amino acids (pyrrolysine and selenocysteine) that can also be specified by the DNA [19, 20], but as in our simple example (Table 1), also from the PTM amino acids, of which thousands have now been described [21, 22]. The only large-scale database for which reliable annotation of PTM amino acids is available is SwissProt, and for this reason (which will be expanded upon below in a separate section) it has been used in this study.

We will use *t*_*i*_ to represent the length in amino acids of the *i* ^*th*^ protein and *a*_*i*_, its unique amino acid count. These two parameters (*t*_*i*_, *a*_*i*_) form a dual by means of which important largely evolution-independent properties can be identified.

Using this pairing for the two proteins above, VG22_BPT2 = (23,6) and PHI3_MYTCA = (45,6). *In information theory then, the protein VG22_BPT2 is entirely reduced to the pair of numbers (23,6)—all other information is discarded.* Since no other information is necessary to demonstrate the Conservation of Information, then by definition it can have little if anything to do with which specific amino acids are present, how often they occur or in what order. As we will see, the distributions of (*t*_*i*_, *a*_*i*_) pairs for every protein considered together, have collective properties inferred by the Conservation of Information which by construction are also independent of which particular amino acids are present, how often they occur or in what order. Reduced to its simplest terms, these properties are global and independent of physicochemical properties in the same way that the general gas equation operates regardless of the specific gas under consideration.

### Conservation of Information: predictions for a system of proteins

The theory is developed in full in [12] and will only be touched upon here. In essence it merges Hartley-Shannon Information theory along with a variational method in statistical mechanics to find the most likely distribution of unique token counts *a*_*i*_ (unique amino acids here) amongst M components (proteins here) with *t*_*i*_ tokens (total amino acids), where i = 1,..,M, whilst conserving both the total number of tokens (amino acids here) T and the total Hartley-Shannon Information Content I, which are respectively defined as

$$\begin{array}{c} T=\sum_{i=1}^{M}t_{i} \end{array}$$ (1)

$$\begin{array}{c} I=\sum_{i=1}^{M}t_{i}lna_{i} \end{array}$$ (2)

Using the method of Lagrange multipliers, the most likely distribution turns out to be given by:-

$$\begin{array}{c} \frac{t_{i}}{T}=\frac{a_{i}^{-\beta }}{Q(\beta )} \end{array}$$ (3)

where *Q*(*β*) is a normalisation constant. This can be interpreted as a probability [23] and we therefore interpret the marginal probability *p*_*i*_ ≡ *t*_*i*_/*T* of appearance of a component with *a*_*i*_ unique amino acids as given by Eq (3). This is scale-independent and does not depend on what the tokens (amino acids here) actually mean in accordance with the underlying model of Hartley-Shannon information. This is intimately associated with the Conservation of Information since this is one of the constraints under which it appears and this is prediction P1. The distribution is a power-law and we note that such distributions are found in many physical and biological systems and have been widely studied [10, 23–29].

A further consequence is that the lengths of proteins *t*_*i*_ in amino acids are uniformly distributed for a fixed amino acid count, [12]. This leads to the conclusion that the average length of a protein is constant for a fixed amino acid count as stated in prediction P2.

Although not pointed out in [12], it can be easily checked by substitution that Eq (3) has a dual solution:-

$$\begin{array}{c} \frac{a_{i}}{A}=\frac{t_{i}^{-1/\beta }}{\sum_{i=1}^{M}t_{i}^{-1/\beta }} \end{array}$$ (4)

where

$$\begin{array}{c} A=\sum_{i=1}^{M}a_{i} \end{array}$$ (5)

Interpreting *a*_*i*_/*A* as a probability as before indicates that the lengths of proteins *t*_*i*_ in amino acids are also distributed as a power-law (which is prediction P3) and that the unique amino acid counts *a*_*i*_ are conditionally uniformly distributed implying by symmetry that the average unique amino acid count is constant for a fixed *t*_*i*_, (which is prediction P4).

It might be asked what is the relationship between this development of theory and the actual occurrence of amino acids in the SwissProt database as shown in Table 3. Fundamental to Hartley-Shannon information is the irrelevance of the meaning of the signs (here amino acids) and since the information content of a protein is based on any amino acid being chosen at any position, it might be thought that there is a built-in assumption of equal probability of occurrence of each of the amino acids at a given position. However, the above development is an *ergodic* theory—it covers all possible system re-arrangements of total size T and total information content I. The system of proteins in SwissProt is just one possible arrangement which has evolved under constraint by the power-law equilibrium position described above. This is exactly analogous to tossing a fair coin 100,000 times and recording the result as just one possible arrangement of all those that might occur. Only in rare cases will an equal number of heads and tails result even though that is the equilibrium position for all possible results of tossing a coin 100,000 times. When only one arrangement is available, we are limited to looking for the footprint of the conservation principle as we do here.

**Table 3:** The amino acids (ignoring any post-translational modification) found in the protein sequences of SwissProt version 13-11, in increasing order of frequency. The total number of amino acids here is 188,892,295.

Letter	Amino Acid	Occurrences
O	Pyrrolysine (Pyr) (Extra)	29
Z	Either Glutamic acid or Glutamine (Glx)	314
U	Selenocysteine (Sec) (Extra)	327
B	Either Aspartic acid or Asparagine (Asx)	344
X	Unknown or ‘other’ amino acid (Xaa)	8665
W	Tryptophan (Trp)	2096837
C	Cysteine (Cys)	2569145
H	Histidine (His)	4365997
M	Methionine (Met)	4625115
Y	Tyrosine (Tyr)	5521997
N	Asparagine (Asn)	7131889
F	Phenylalanine (Phe)	7446033
Q	Glutamine (Gln)	7542048
P	Proline (Pro)	9047014
T	Threonine (Thr)	9860719
D	Aspartic Acid (Asp)	10479769
R	Arginine (Arg)	10635595
K	Lysine (Lys)	10721971
S	Serine (Ser)	11191510
I	Isoleucine (Ile)	11476704
E	Glutamic Acid (Glu)	12999633
V	Valine (Val)	13225777
G	Glycine (Gly)	13558574
A	Alanine (Ala)	15774048
L	Leucine (Leu)	18612241

Finally, we note that prediction P4 indicates the unique amino acid count as a real-valued variable whereas in a protein it is of course an integer. The average value is only a statistical model that nevertheless (as we shall show) displays an extraordinary linearity at integral values, just as it does at those points which do not correspond to integer values.

### 0.1 The SwissProt protein sequence database

The essential trade-off in current protein sequence databases is between quality and quantity. The highest quality of curation is achieved manually, but manual curation is a strong constraint on quantity because it is so much slower than machine annotation. As a result, SwissProt [18] with its emphasis on manual curation is much smaller than for example, TrEMBL, but it has several advantages for our analysis; it is probably as accurate as can currently be achieved because of the strong emphasis on manual curation, and its PTM documentation is the best available through its association with Selene [22].

PTM are an important factor in information theory because of their impact on the available amino acid choices, i.e. the potential size of the unique amino acid count. Without PTM amino acids, the unique amino acid count for any protein would be constrained to the genetically-encoded amino acids and thus the inclusion of PTM amino acids hugely expands the possible values of the unique amino acid counts for a protein and as a result greatly strengthen the significance of the statistical analyses performed here. Even though the SwissProt database has many fewer entries than e.g. TrEMBL, the dataset is certainly large enough to provide high levels of significance, as will be shown by our analyses. Taking into consideration the strengths and weaknesses of each database we selected SwissProt (release 13-11) for this study, acknowledging that only certain of the biases present in this dataset could be corrected for.

However, 3 particular possible sources of bias in SwissProt were identified and investigated as follows.

- *Methionine/ (N-formyl methionine) start codons.* These amino acids, encoded at the N-terminus of proteins, are removed by cells if N-terminal processing is required to produce the mature functional protein. It is reasonable to assume that all eukaryotes and archaea should have methionine as the first amino acid of their proteins and that all bacterial proteins should have N-formyl methionine as their first amino acid. The distributions actually found in SwissProt are shown in Table 4. As can be seen the vast majority of proteins (527,843 out of 541,762) of all four domains of life have methionine (N-formyl methionine) as their first amino acid as expected. Of the remaining 13,919, methionine occurs elsewhere in the sequence for 8,471 proteins, thus leaving the unique amino acid count unaltered since this depends only on methionine occurring at least once somewhere in a eukaryotic or archaeal protein sequence. This leaves only 5,448 out of 541,762 proteins (1%) for which the unique amino acid count would be increased by 1 if methionine (N-formyl methionine for bacterial proteins) was registered in the first position. This was treated by computing the (*t*_*i*_, *a*_*i*_) pairs as they are found in SwissProt 13-11 and comparing the results with those obtained when methionine (or N-formyl methionine for bacteria) was added as the N-terminal residue for the 5,448 proteins missing methionine (N-formyl methionine). The effect of this addition on the unique amino acid counts for all proteins is everywhere less than 0.4% and in most cases, much smaller still. This was found to have a negligible effect on the results of the analyses, (Table 5 M-fixed), where the power-law tail of the distribution of unique amino acid count remains essentially unchanged and emphatically linear.
- *Signal peptides.* These are sequences (typically N-terminal stretches of up to approximately 30 amino acids) that direct proteins to the endoplasmic reticulum (in eukaryotes) and permit membrane insertion of proteins or their entry to the secretory pathway. The signal peptide is typically cleaved in the process and thus is missing from certain experimentally-derived protein sequences. Signal peptides are annotated in SwissProt, and the simplest way of assessing how much of an impact they have on the size of proteins and the total unique amino acid count, is to calculate all of the results both with and without the signal peptides included. All 541,762 proteins were processed, first each with its peptide sequences included and then each with its peptide sequences excised. The effect is somewhat larger than that due to the selective methionine insertion described above and reduces the protein counts by around 2.9% but this still has a negligible effect on relative unique amino acid counts and the power-law tail, (Table 5. No peptides). The effect of excising these peptide sequences was also checked on the protein length distributions and average protein lengths and found to be similarly negligible.
- *Ambiguities in the unique amino acid count.* It is non-trivial to extract the unique amino acid count from SwissProt even using the Selene PTM lists, and ambiguities in unique amino acid counts proved the most difficult to model, necessitating Monte-Carlo methods to assess the sensitivity. (The process used is described in detail in the Methods section.) The effects of ambiguities in the unique amino acid count were estimated using this method and shown to have a negligible effect on the analyses, (Methods and Table 5, Monte Carlo). This approach differs from the treatment of the previous two sources of bias (above) in that it randomly perturbs the means by which the unique amino acid count is calculated within certain bounds whereas the previous 2 methods actually modify the sequences appropriately in a non-random way before calculating the unique amino acid counts.

**Table 4:** Distribution of methionine and N-formyl methionine (M) initiated proteins in SwissProt 13-11.

Domain	Total proteins	M-initiated	N-formyl M initiated
Archaea	19,063	19,009	0
Bacteria	329,256	328,038	31
Eukaryota	177,020	164,798	85
Viruses	16,423	15,998	0

**Table 5:** Analysis of the Impact of Potential Bias in the SwissProt dataset. Bias was assessed in terms of impact on the fit statistics for power-law behaviour in the tail of the unique amino acid count distribution, which is addressed in detail in the following sections as prediction P1, (see also Fig 1A). Datasets *P*_*sub*_ (*a*_*i*_ = 20 to 30), *P*_*exp*_ (*a*_*i*_ = 20 to 31) and *P*_*all*_ (*a*_*i*_ = 20 to 37), were analyzed as extracted from SwissProt and are described in Methods. The fit statistics for the power-law tail were then compared with the equivalent fit statistics on datasets corrected for potential bias in treatment of the initiating methionine (M-fixed), inclusion or exclusion of signal peptides (No peptides) and Monte-Carlo exploration of the ambiguity of unique amino acid counts as described in Methods. The fit statistics are remarkably resilient with respect to all three different possible sources of bias, and the robust linearity of the power-law tail in all conditions is emphasized by the high values of the adjusted *R* ^2^.

Dataset	Slope	Std. error	Adj *R* ^2^	F	DF	p
*P*_*sub*_	-24.139	0.6466	0.962	251	9	(7.028) × 10^−08^
*P*_*exp*_	-22.914	0.2238	0.995	2027	9	(6.526) × 10^−12^
*P*_*all*_	-16.342	0.5040	0.974	588	15	(1.908) × 10^−13^
*P*_*exp*_ M-fixed	-22.917	0.2242	0.995	2021	9	(8.488) × 10^−12^
*P*_*all*_ M-fixed	-16.343	0.5041	0.974	588	15	(1.911) × 10^−13^
*P*_*exp*_ No peptides	-29.378	0.1986	0.997	2317	7	(4.367) × 10^−10^
*P*_*all*_ No peptides	-19.901	0.2040	0.996	2638	11	(1.873) × 10^−14^
*P*_*exp*_ Monte Carlo	-22.984	0.2287	0.995	1954	9	(7.754) × 10^−12^
*P*_*all*_ Monte Carlo	-16.355	0.5050	0.973	587	15	(1.940) × 10^−13^

We proceeded therefore with the analysis of the whole SwissProt 13-11 database as is, (it contains sequences for a total of 13,041 organisms, comprising 541,762 proteins) in the knowledge that at least the identified potential sources of bias had a negligible effect on the results presented below.

### Predictions implied by Conservation of Information

The mathematical argument underlying these predictions, following [12], has been presented above. In summary, these predictions assert that proteins, considered collectively and *without regard to species or domain of life*, should show the following global properties

- P1) The distribution of numbers of proteins should asymptote to a power law in unique amino acid count,
- P2) Proteins with a given unique amino acid count should have the same average length,
- P3) The distribution of protein lengths should asymptote to a power law,
- P4) Proteins with a given range of lengths should have the same average unique amino acid count,
- P5) The distributions are scale-independent and should be present in subsets of the data.

In the following sections we examine to what extent these 5 predictions are supported. The results demonstrate that these predictions hold to a very high degree of significance, (the largest p-value we found in any of our analyses, including those modelling the possible sources of bias described earlier was 7.028 ×10^−8^), strongly supporting the proposition that Conservation of Information is a principle that guides the global structure of the system of proteins analyzed here.

## Methods

### Statement of Reproducibility

The complete means to reproduce all of the results of this paper including all source code, R graphics scripts, R statistics scripts, shell scripts, makefiles along with detailed build and operational instructions are available for public download at http://leshatton.org/ following the recommendations in [30].

### Software compliance

The analysis software is a mixture of perl, C and R scripts for statistical analysis and plotting. The C source code is compatible with the current ISO C standard ISOC2011:9899 and the perl scripts run on all recent versions of perl, (version v5.16.2 was used). Only one CPAN (http://cpan.org) package is used, LWP::UserAgent to download the appropriate version of SwissProt, in this case version 13-11, and this was already embedded in the download example script supplied on the SwissProt site.

In principle this can all be assembled and run in a Windows environment but the whole of this project was implemented and carried out using Linux systems, specifically a standard Open SuSE12.3, (http://opensuse.org/) release, although any modern Linux installation will suffice since no special components were used.

### Downloading and unpacking

The SwissProt dataset was downloaded in the appropriate FLAT file format using a script supplied at http://uniprot.org/faq/28#downloading. The format used was ‘txt’ and the complete set of protein sequences were downloaded for SwissProt release 13-11. The supplied script downloads a single SwissProt distribution file uniprot_sprot.dat of size 2.7Gb. This file was then unpacked into four domains, archaea, bacteria, eukaryota and viruses by parsing the OC line in the distribution and their individual species by parsing the ID line using a hand-crafted perl program.

This produced a .fasta file for each species containing all the proteins for that species. In all, 195 archaean species, 2807 bacterial species, 7475 eukaryotic species and 2564 viral species were extracted, totalling 13,041 species and comprising a total of 541,762 proteins.

For example, the FT lines and the protein sequence itself are shown in this format for the bacterial taxon AMISM in Table 6.

**Table 6:** Example FT and SQ records from an extracted .fasta file.

FT	PEPTIDE	1	14	Amythiamicin A/B.
FT				/FTId = PRO_0000368029.
FT	PEPTIDE	1	12	Amythiamicin C/D.
FT				/FTId = PRO_0000368030.
FT	MOD_RES	3	3	N4-methylasparagine.
FT	MOD_RES	12	12	Cyclo[(prolylserin)-O-yl] cysteinate; in
FT				form C.
FT	MOD_RES	12	12	Cysteine methyl ester; in form D.
FT	MOD_RES	14	14	Proline amide; in form A and form B.
FT	CROSSLNK	1	11	Pyridine-2,5-dicarboxylic acid (Ser-Ser)
FT				(with C-10).
FT	CROSSLNK	1	10	Pyridine-2,5-dicarboxylic acid (Ser-Cys)
FT				(with S-11).
FT	CROSSLNK	1	2	Thiazole-4-carboxylic acid (Ser-Cys).
FT	CROSSLNK	3	4	5-methylthiazole-4-carboxylic acid (Asn-
FT				Cys).
FT	CROSSLNK	5	6	Thiazole-4-carboxylic acid (Val-Cys).
FT	CROSSLNK	8	9	Thiazole-4-carboxylic acid (Val-Cys).
FT	CROSSLNK	9	10	Thiazole-4-carboxylic acid (Cys-Cys).
FT	CROSSLNK	11	12	Thiazole-4-carboxylic acid (Ser-Cys).
FT	CROSSLNK	12	13	Oxazoline-4-carboxylic acid (Cys-Ser); in
FT				form A.
FT	UNSURE	4	4	C or T.
SQ	SEQUENCE	14 AA;	1365 MW;	3EB862761A777DC8 CRC64;
	SCNCVCGVCC			SCSP

### Extracting unique amino acid counts in Selene

In spite of its pivotal role in the Conservation of Information, extracting the unique amino acid counts proved somewhat difficult with the Selene datasets. The problem can be described as follows. Let X be the unique amino acid count in a protein before PTM, P be the number of PTM in the protein, and Y be the unique amino acid count after PTM in the protein. The best available value of P is derived from proteins in the set *P*_*exp*_ and X can be extracted simply and unambiguously from the headers in the FLAT format files in SwissProt. Unfortunately, Y is not so readily available. It might be thought simplistically that

$$\begin{array}{c} X+P=Y \end{array}$$ (6)

In other words, the PTM simply supplement the existing unique amino acid count for a particular protein. This would assume however that PTM has no effect on the original unique amino acid count, but it is possible to construct sequences for which this would not be true. For example, if an amino acid occurs once in a protein sequence and is post-translationally modified, the unique amino acid count remains the same, violating Eq (6), (it would give X + 1 = X). Given that we wish to use *P*_*exp*_ as the definitive source for P for each protein, it was important to estimate how often Eq (6) is violated to estimate the magnitude of any resulting error. We therefore wrote specialist software included in the reproducibility deliverables to extract a controlled subset of PTM for each protein, *P*_*sub*_ say, from the SwissProt database from which X, P (taken from *P*_*sub*_) *and* Y could be directly measured for each protein against Eq (6).

This was done as follows.

The length in amino acids and the unique amino acid counts (X) for each protein was extracted from the FT and SQ records of the.fasta file format shown above. The modified residue sites including the non-standard amino acids ‘U’ for selenocysteine [19] and ‘O’ for pyrrolysine [20], were then extracted from the FT header lines and the corresponding sites modified. For reasonable tractability, this was restricted to single sites only, (start and end column identical) and further restricted to FT records containing the FASTA keywords MOD_RES, LIPID, CARBOHYD and DISULFID. No more than one modification was allowed per site. This yielded P and the unique amino acid count was then recalculated after (Y). The extracted lengths of the sequences were checked where there was overlap [31] and a number of proteins were also selected and manually checked for accuracy on the residues to confirm the results obtained by the software.

The results are shown in Table 7 which shows that the vast majority of PTM modifications (> 99%) using the controlled *P*_*sub*_ subset increase the unique amino acid count in line with Eq (6). In order to estimate the potential impact on using the more comprehensive Selene PTM classification *P*_*exp*_, it was assumed that the same distribution would hold for this also. To stress test this assumption, a Monte-Carlo model was carried out using P taken from *P*_*exp*_ and randomly perturbed by the distribution defined by Table 7 to calculate Y. The result of this suggested that breaches of Eq (6) had a negligible effect on the results shown earlier, (Table 5, Monte Carlo).

**Table 7:** A check on the non-independence of PTM counting on the unique amino acid count carried out on *P*_*sub*_ as described in Methods, Extracting unique amino acid counts in Selene.

(X+*P*_*sub*_)-Y	Number	Percent
0	537999	99.31%
1	3545	0.65%
2	208	0.04%
3	9	0.00%
4	0	0.00%
5	1	0.00%

It is perhaps worth noting that in spite of the extensive database of documented PTM amino acids [21, 22, 32], the largest unique amino acid count found in our *P*_*sub*_ subset was 30 whilst the largest in the *P*_*exp*_ dataset is only 31, (compared with 37 for the non-experimentally verified Selene dataset).

As a result, the *P*_*exp*_ dataset was used as the definitive source of PTM modifications assuming that Eq (6) remained true. By merging the sequence extracted from the SQ records as above with the PTMs annotated in the Selene file byidexperimental.txt from http://selene.princeton.edu/PTMCuration/, the unique amino acid counts were then extracted.

It should also be noted that this paper identifies a close analogy between the information properties of software [12] and those of proteins. To add further substance to the methodology here, the software written to analyse the former (which is also available in full at http://leshatton.org/ and is written in ISO C 2011:9899) was redesigned from scratch in a different programming language, perl, to reduce the risk of common mode software failure in the methods of analysis [33]. The information properties extracted for these two very different regimes were nevertheless identical. Both sets of analysis software are freely downloadable for open and thorough scrutiny of the overall methodology.

### Statistical analysis

All statistical analysis was carried out using R (http://r-project.org/) scripts included in the reproducibility deliverables. The analysis requirements were relatively simple and only the following statistical functions were used:- lm() for linear modelling and prop.test() for multi-proportion testing. In each case, the relevant statistic, adjusted R-squared or chi-squared was quoted along with the p-value. No p-value was found in any of the tests exceeding 7.028 ×10^−8^ indicating that the probability of finding a more unlikely dataset by chance was exceedingly small.

## Results

### The distribution of number of proteins asymptotes to a power law in the size of the unique amino acid count (P1)

**Fig 1:**
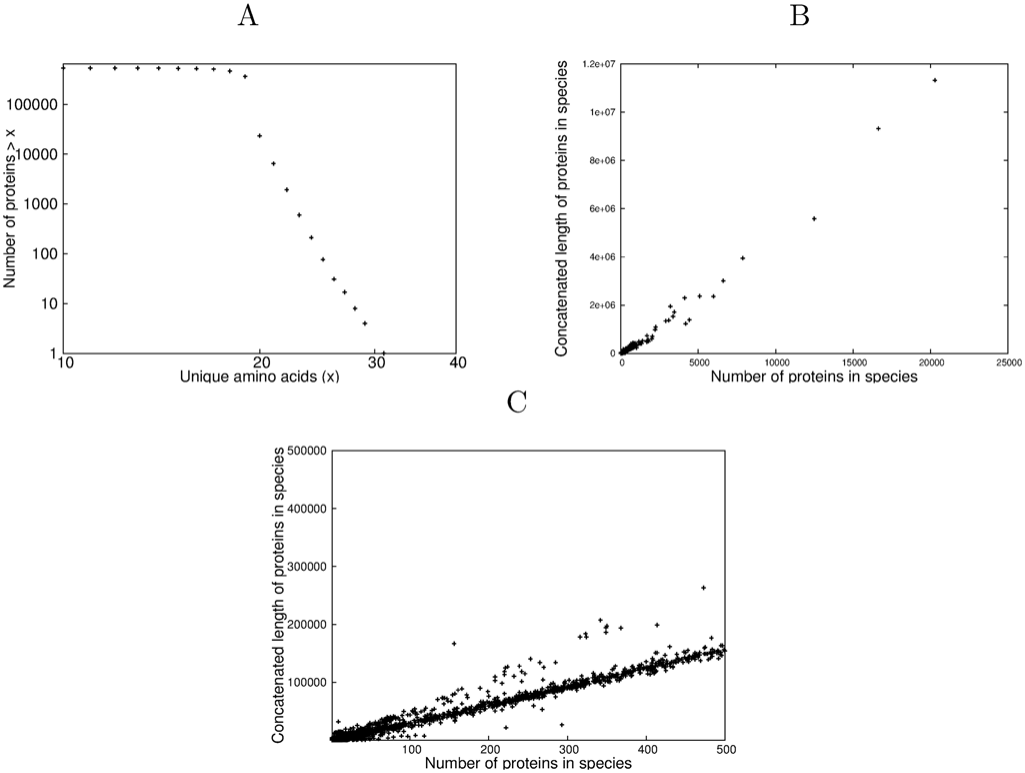
Unique amino acid distribution and average protein length. A Cumulative distribution function of unique amino acid count occurrence in proteins. The analysis encompassed all archaeal, bacterial, eukaryotic and viral proteins that were combined into the experimental PTM *P*_*exp*_ subset [22]. B A plot of number of proteins against total length of those proteins for each species in SwissProt for unique counts from 1-30 amino acids. Each data point is one of the 13,041 species analysed C An expanded plot of the data in the bottom left hand corner of Fig 1B for species with less than 500 proteins, showing sub-structure.

Fig 1A shows the log-log cumulative distribution plot of the number of proteins against the size of their unique amino acid count *a*_*i*_ for the dataset *P*_*exp*_, the set of proteins extracted from Selene that have experimentally verified PTM [32]. On such a plot, a power-law relationship appears linear [10]. The distribution shows a well-developed power-law tail $\;a_{i}^{-\beta }$, where *β* is the slope, for unique amino acid counts between 20 and 30 inclusive—the linearity of the relationship is emphatic, with adjusted *R* ^2^ = 0.995, *β* = −22.914 and p = (6.526) × 10^−12^), Table 5. These data are drawn globally from all domains of life and illustrate behavior that is therefore independent of all properties of proteins except for the size of their unique amino acid counts.

### Proteins with a fixed unique amino acid count have the same average length (P2)

To show this in its simplest form, for each of the 13,041 species considered in this analysis, the number of proteins *n*_*p*_ in each species was extracted along with their total length *l*_*p*_. The average length of proteins in a species is then given by *l*_*p*_/*n*_*p*_. Conservation of Information predicts that the lengths of proteins are uniformly distributed for a fixed unique amino acid count, so the average length, *l*_*p*_/*n*_*p*_ should be constant across all species and a plot of *l*_*p*_ against *n*_*p*_ for each species in the analysis should therefore be linear with a slope of *l*_*p*_/*n*_*p*_. This allows us to measure the quality of the linearity again by linear modelling using the adjusted *R* ^2^ and associated p-value.

**Fig 2:**
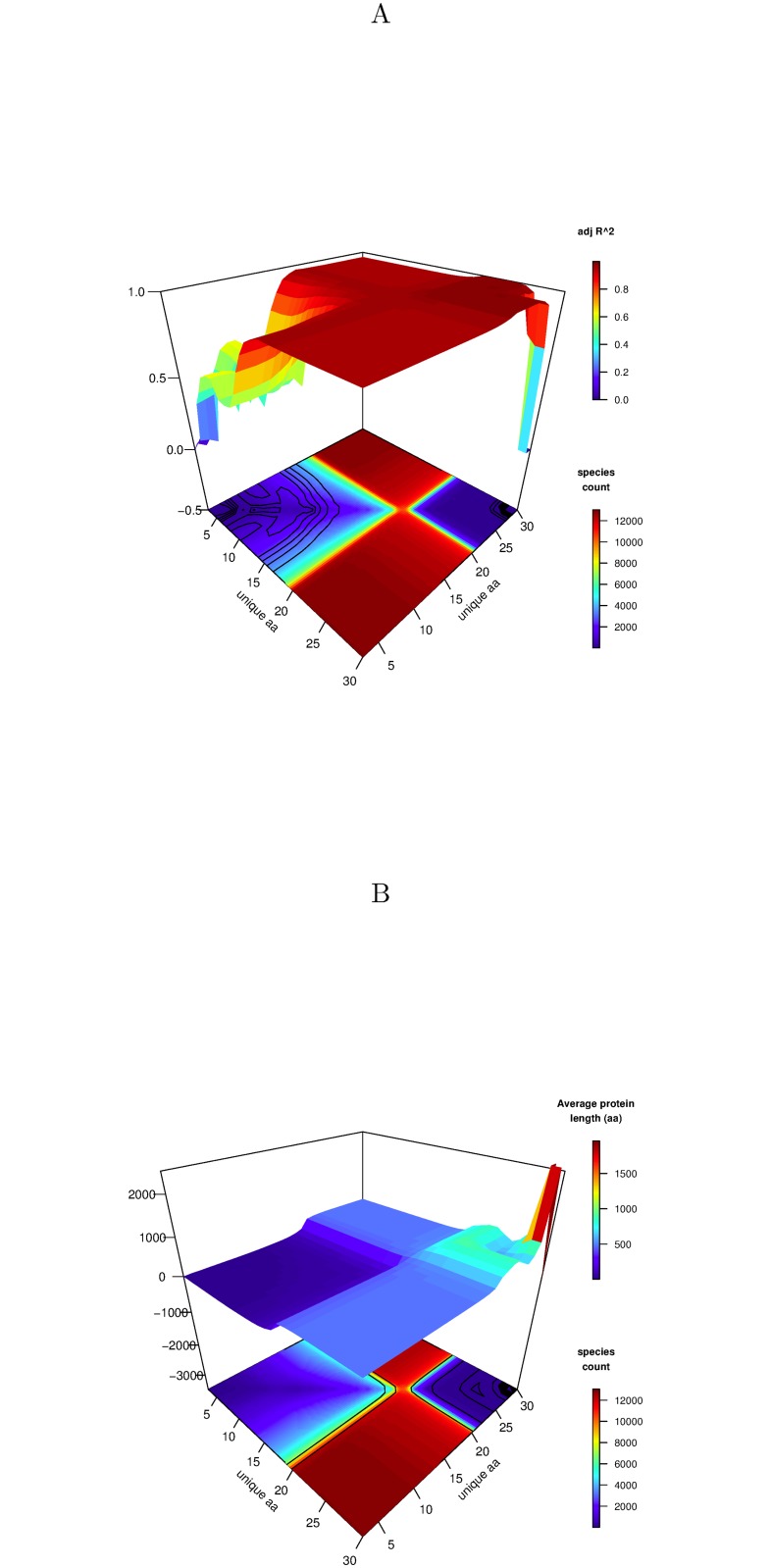
Adjusted *R* ^2^ and average protein length distribution with unique amino acid count. A A plot of the adjusted *R* ^2^ against the range of unique amino acid counts. Each point on the surface corresponds to the *R* ^2^ match of all species for those proteins which fall in the unique amino acid count specified. The lower part of the plot contours the number of contributing species. B A plot of the average protein length for all species against the range of unique amino acid counts. The lower part of the plot contours the number of contributing species.

Fig 1B and 1C demonstrate the linearity of *l*_*p*_/*n*_*p*_ for all the proteins in SwissProt where each datapoint corresponds to a species. (Fig 1C is an expanded view of the plot for those species with fewer than 500 proteins in the database). Strictly speaking, P2 refers to a specific unique amino acid count but the linearity is extremely well established, (adjusted *R* ^2^ = 0.967, p < (2.2) × 10^−16^, calculated for the full data set as in Fig 1B), even though all proteins with unique counts between 1 and 30 were included. To assess the linearity over all possible sub-ranges of unique amino acid count, the adjusted *R* ^2^ was computed on an (x,y) grid as shown in Fig 2A. For example, the point x = 15, y = 25 shows that for all proteins with a unique amino acid count between 15 and 25, the adjusted *R* ^2^ value was in excess of 0.9 as it is across most ranges. The lower part of this plot shows the number of contributing species for the same sub-ranges of unique amino acid count. As can be seen, the value of adjusted *R* ^2^ remains high until the species count is low, indicating the robustness of this result.

We note observationally that the average length of proteins changes with the unique amino acid count as can be seen in Fig 2B by following the diagonal across the upper surface from left to right, where the average length is plotted here on an (x,y) grid of ranges of unique amino acid count. As can be seen, there is a subtle and increasing relationship between the average protein length and the unique amino acid count. The lower part of Fig 2B shows the corresponding number of contributing species.

This is relevant to Fig 1C where there is some evidence of fine structure. This is partly due to the fact that all proteins with fixed unique amino acid counts between 1 and 30 are displayed for numerical strength and partly because there is evidence that eukaryota and viruses both show larger departures from the equilibrium position defined by Conservation of Information than the archaea and bacteria, which we ascribe to evolutionary pressures, [34–38]. Conservation principles act as constraints at the global level rather than straitjackets at the local (in this case evolutionary) level. The fine structure observed in Fig 1C also has relevance to the relationship of average protein length to unique amino acid count. Although the data are not shown here, the fine structure in Fig 1C is interpreted as resulting partially from the fact that that eukaryota and viruses both show larger departures from the equilibrium position defined by Conservation of Information than the archaea and bacteria, and partially from the inclusion in the display (for numerical strength) of all proteins with unique amino acid counts between 1 and 30.

### The distribution of protein lengths asymptotes to a power law (P3)

**Fig 3:**
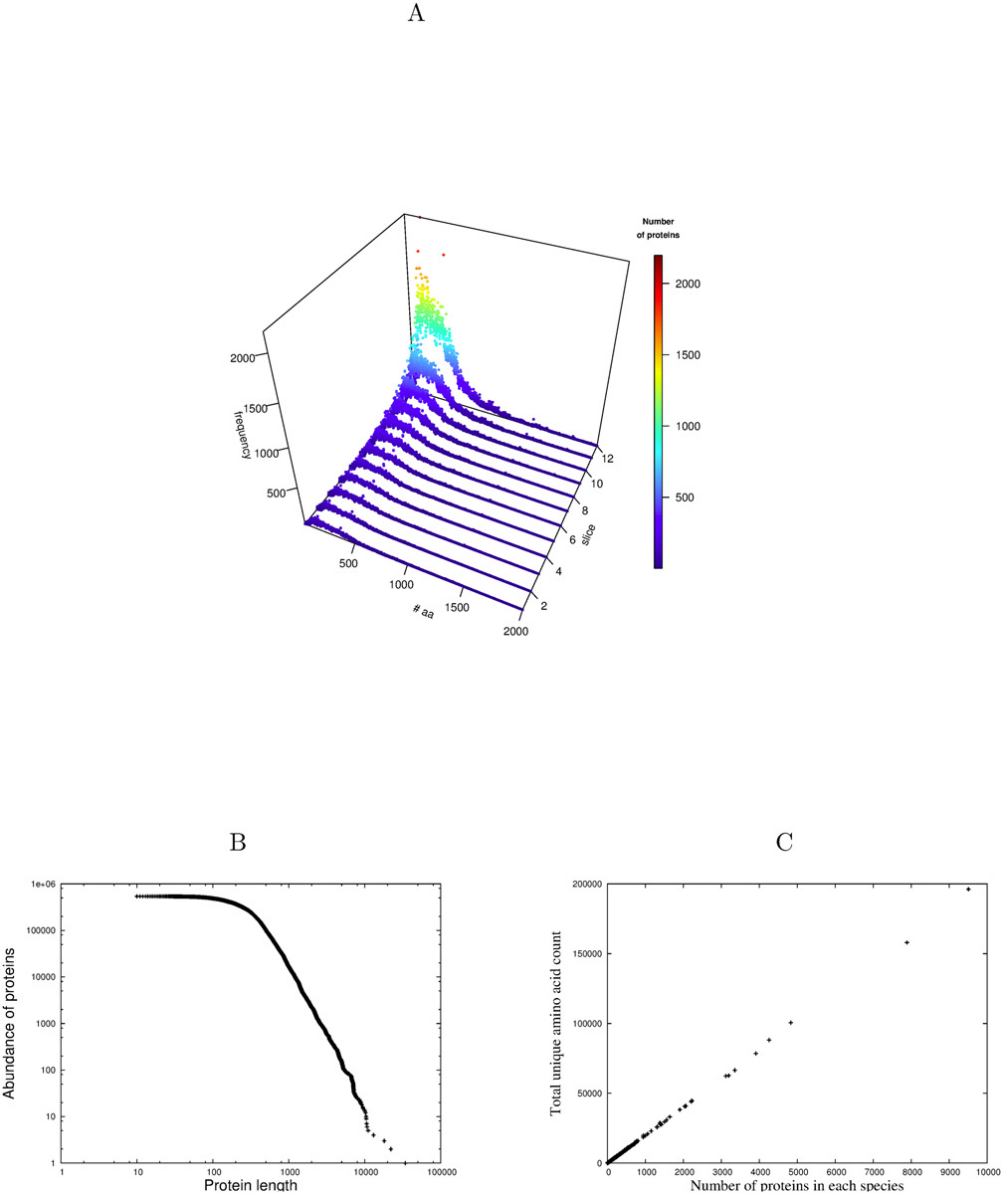
Power-law distribution of protein lengths. A The emergence of the protein length distribution with randomly increasing subsets of data from the full SwissProt database. B The length distribution of all proteins plotted as a log-log cumulative distribution function illustrating the emphatic power-law tail. C A plot of number of proteins against total length of their unique amino acid counts for each species in SwissProt for protein lengths from 100–500 amino acids. Each data point is a species.

A plot of the occurrence of proteins against their length (defined as the total number of amino acids in their sequence) is shown in Fig 3A. Successively larger random samples of proteins were taken from the full SwissProt database (*P*_*all*_) showing the emergence of the final distribution as increasing amounts of data are plotted. The fully populated distribution (slice 12) when plotted as a log-log cumulative distribution function, Fig 3B, demonstrates emphatically the predicted power-law tail, (between *t*_*i*_, proteins of total length between 300 and 10,000 amino acids inclusive, the adjusted *R* ^2^ = 0.997, with p < (2.2) × 10^−16^).

### Proteins with a fixed length have the same average unique amino acid count (P4)

**Fig 4:**
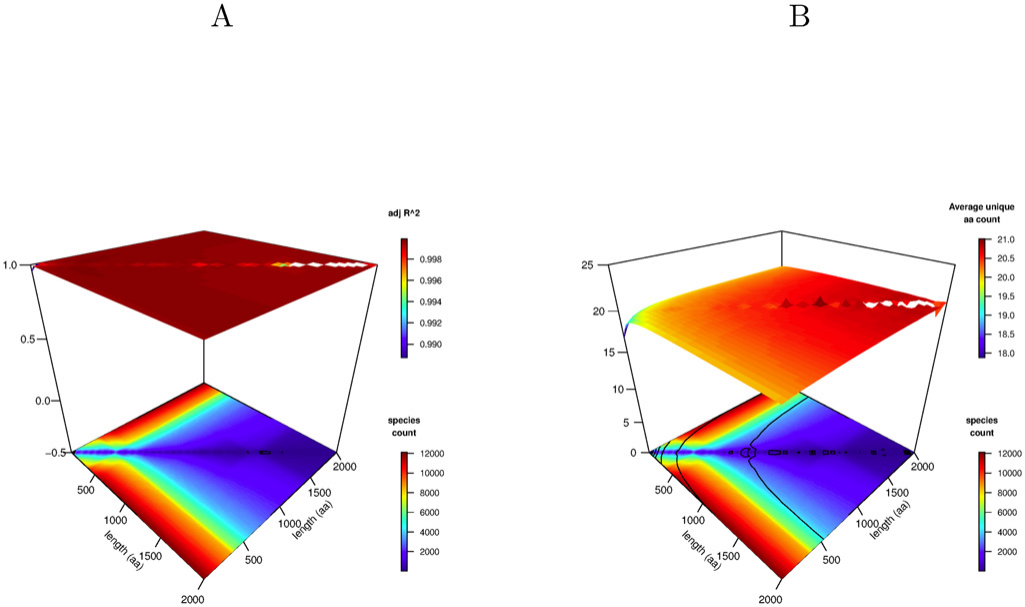
Average unique amino acid count distribution. A A plot of the adjusted *R* ^2^ against protein length for the match between number of proteins and their total unique amino acid count for all species. Each point on the surface corresponds to the *R* ^2^ match of all species for those proteins whose lengths fall in the specified length range. The lower part of the plot contours the number of contributing species. B A plot of the average protein length for all species against the range of unique amino acid counts. The lower part of the plot contours the number of contributing species.

Since in general few proteins will have a particular length, we must choose a range of lengths which encompasses sufficient qualifying proteins whilst preserving the nature of the prediction. As was the analogous case with P2, to show this prediction in its simplest form, for each of the 13,041 species in SwissProt and a fixed range of lengths, the number of proteins *n*_*p*_ in each species was extracted along with the sum of their unique amino acid counts *l*_*a*_. If the unique amino acid count is distributed uniformly as predicted, the average unique amino acid count *l*_*a*_/*n*_*p*_ should be constant across all species and a plot of *l*_*a*_ against *n*_*p*_ for each species should be linear. Fig 3C illustrates this relationship between average unique amino acid count and protein length for all proteins of length between 100 and 500 amino acids. In spite of this relatively wide range of qualifying protein lengths, the linearity is truly extraordinary, (adjusted *R* ^2^ = 0.999, p < (2.2) × 10^−16^), emphasizing once again the important organizing role that the unique amino acid count plays in the structure of proteins, completely independently of the physicochemical nature of those amino acids or the domain of life in which the proteins have evolved. This analysis is extended on a grid over protein length ranges between 50 and 2000 amino acids in Fig 4A and the adjusted *R* ^2^ plotted for each range. It is again remarkable that across most of this grid, the adjusted *R* ^2^ exceeds 0.998.

It is very clear from Figs 3C and 4A that, in so far as the set of proteins in SwissProt is representative, an archaean protein of, for example length 100 amino acids, has the same average unique amino acid count as a bacterial, eukaryotic or viral protein of the same length emphasizing the taxonomy-independent properties of the unique amino acid count. By extension, longer proteins of any species will on average have a larger unique amino acid count independently of their taxonomy.

The dependence of the unique amino acid count on the protein length is shown in Fig 4B where it is plotted on a grid of ranges of protein length. Looking along the plane of the upper surface from left to right, the unique count is observed to increase gently with protein length.

### The distributions are scale-independent and are present in subsets of the data (P5)

**Fig 5:**
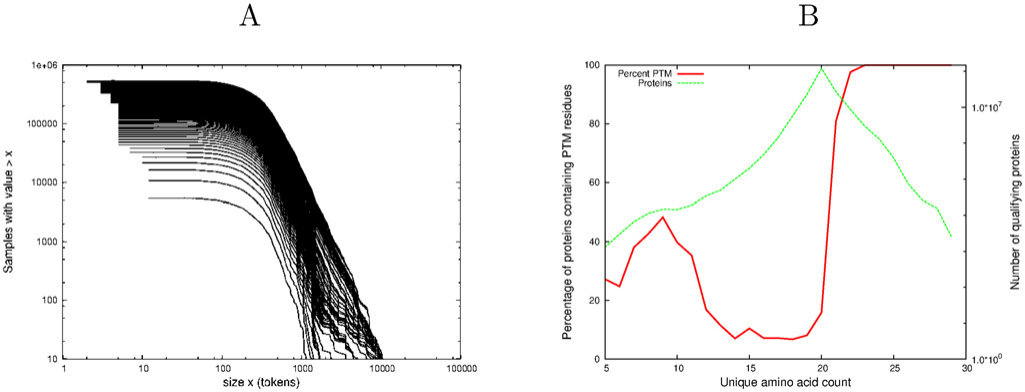
Persistency and PTM occurrence rate. A Cumulative distribution function plots of length distribution for increasingly large random subsets of the proteins present in SwissProt illustrating that power-law behaviour is persistent. B The percentage of proteins which contain PTM amino acids, and the number of proteins, as a function of unique amino acid count.

It might be thought that power-law behavior is an emergent property but in fact the scale-independence of the underlying mathematics predicts that it is a persistent property present throughout a system as it grows, [12]. Fig 5A simulates this by taking 100 random selections of the SwissProt protein length data ranging from 1% of the data (on the inside), to 100% (on the outside), which are shown demonstrating the essentially self-similar behavior devolving from this scale-independence.

## Discussion

### Global conservation constraints allow evolutionary departures from equilibrium

We conclude that the outcome of experimental tests of predictions derived from the Conservation of Information strongly suggests that this principle constrains the global structure of the system of proteins examined, and by implication sets boundaries on the structure of proteins within which natural selection can act. However, these global constraints do not preclude the presence of local effects, as manifest in differences between taxa and substructure within the data. These have been noted by others and merit discussion here in the light of our findings.

First we note the presence of fine structure in the distribution of average protein lengths. Previous reports have considered average protein (or gene) lengths from a taxonomic perspective, observing a) constancy within domains of life but departures across them [34, 35, 39]; b) evolutionary effects from functional constraints [40]; and c) length distributions in different phylogenetic groups measuring specific correlations with individual named amino acids, [31].

**Fig 6:**
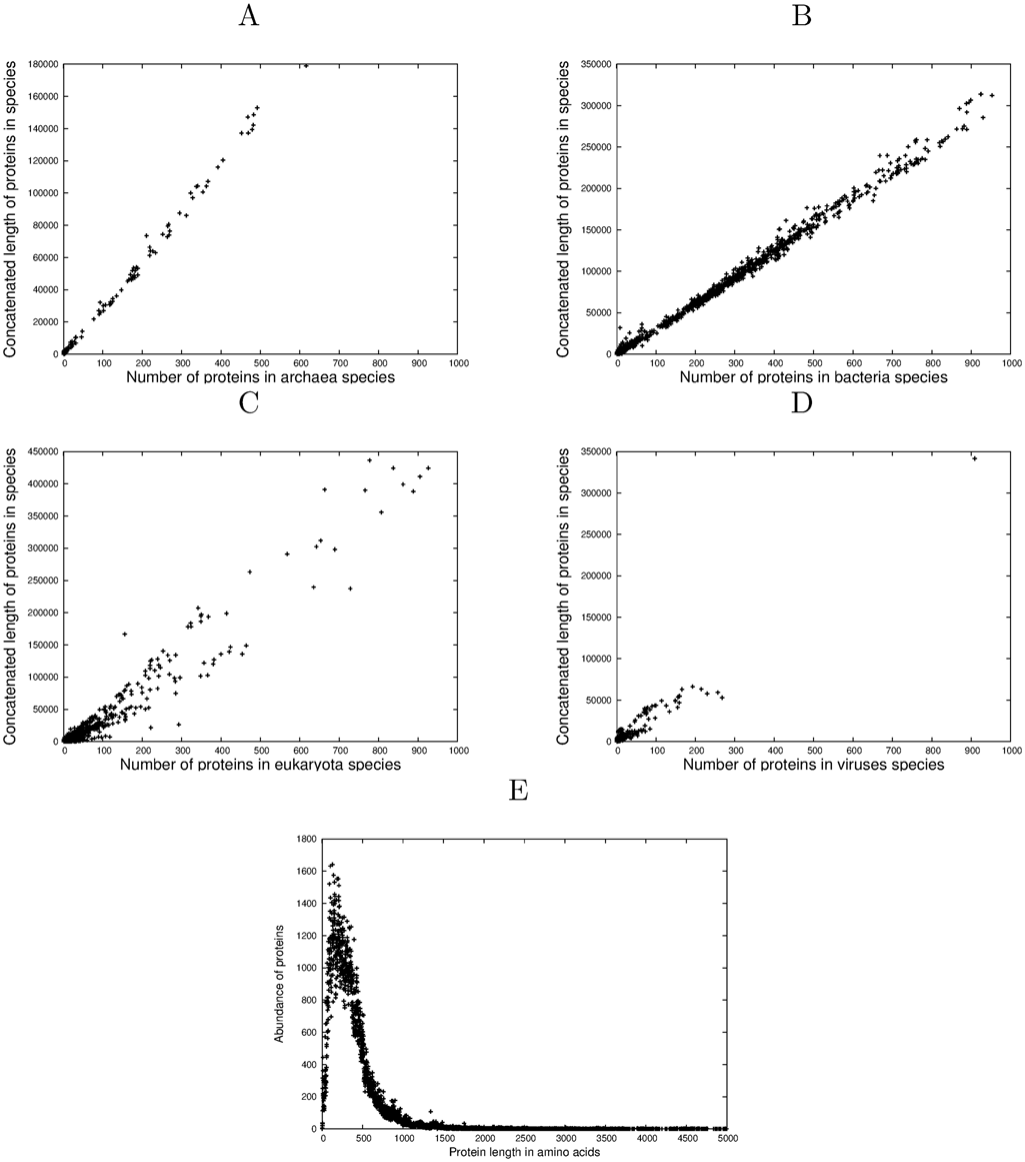
Average protein lengths in the Four Domains of Life and complete distribution. A Average length of proteins for archaean species. B Average length of proteins for bacterial species. C Average length of proteins for eukaryotic species. D Average length of proteins for viral species. E The length distribution of all proteins in the analyzed SwissProt database, release 13-11.

The length data for all four domains represented in SwissProt are shown as Fig 6A–6D for proteins up to 1000 amino acids, and confirm in principle previous reports [34, 35, 39]. For archaea and bacteria, average protein length is very highly conserved. For archaea, Fig 6A, the average length is 295 amino acids (adjusted *R* ^2^ 0.997, *p* < 2.2 × 10^−16^). For bacteria, Fig 6B, the average length is 314 amino acids (adjusted *R* ^2^ 0.997, *p* < 2.2 × 10^−16^), the same quality of fit as for the archaea. Turning to the eukaryota, Fig 6C, there appears more substructure than is the case with the archaea and bacteria, (adjusted *R* ^2^ 0.941, average length 435 amino acids, *p* < 2.2 × 10^−16^). If the data for the eukaryotic species with > 1,000 proteins in SwissProt are included in the analysis, the average protein length for eukaryota increases to 522 amino acids. Fig 6D similarly shows fine structure imposed on a generally linear relationship for viral proteomes, (adjusted *R* ^2^ 0.927, average length 345 amino acids, *p* < 2.2 × 10^−16^). This is the worst (but nevertheless convincing) of the linear fits and we note that viruses present an extreme case of evolutionary diversification; the hosts on whose biochemical machineries they depend range across all domains of life, and viral proteomes range in size from around 10 proteins for the smallest viruses such as poliovirus to over 1,000 proteins for the huge pandoraviruses [36].

Given these strong correlations and highly significant p-values, we interpret these results as strong evidence of a global tendency to constant average length (the equilibrium position) that constrains the effects of the local evolutionary pressures that are visible in the fine structure.

Second, the presence of local evolutionary departures from the global distribution raises a further interesting question. The amino acid repertoire of life is made up of the 22 amino acids which can be specified by the DNA supplemented by the thousands of PTM amino acids. Is there any evidence to suggest that shorter proteins, which are necessarily associated with a smaller unique amino acid count, are under greater evolutionary pressure to supplement the DNA-specified amino acids with PTM amino acids? In other words, is there a relative over-abundance of PTM amino acids in smaller proteins (i.e. those with lower unique amino-acid counts)? The SwissProt dataset analyzed here suggests that this is indeed the case as can be seen in Fig 5B. A multi-proportion test on unique amino acid counts (6,7,8) and (14,15,16) sampling the lower and middle ranges respectively, emphatically rejects the null hypothesis that the proportions are the same (chi-squared = 3158, p < (2.2) × 10^−16^).

### Incompleteness is a significant concern in protein curation

It is important to note that there is considerable debate about the undercounting of PTM amino acids, particularly with respect to the frequency and extent of glycosylation. Several studies suggest that glycosylation is very common, occurring perhaps in the majority of proteins across all domains of life [37, 38]. This position has consolidated with recent advances in techniques (primarily liquid chromatography and mass spectrometry) for the experimental detection of glycopeptides in both their sensitivity and capacity for high-throughput analysis [41–44]. As noted by Thaysen-Andersen and Packer [42] these advances are enhancing “the depth and coverage of the glycoproteome” and “…..it is clear that only the top of the glycoproteome ‘iceberg’ is covered to date.” Furthermore, experimental validation of *in silico* predictions of PTM is still necessary [45]. We conclude that the degree to which protein PTM occurs is likely to be significantly under-counted, and that the full extent and diversity of glycosylation in particular remains to be uncovered.

This will be an interesting challenge for models based on information theory as data improves in both completeness and accuracy. However, despite this and other measurement problems in protein databases, the fact that the operation of Conservation of Information is still clearly evident in the degree of significance reported for each of the above predictions P1)—P5), points to the robustness of this conservation principle. The extraordinary linearity of the average unique amino acid count across all species (P4) as evidenced by Fig 3C is particularly noteworthy.

### The distribution of Post Translational Modifications

**Fig 7:**
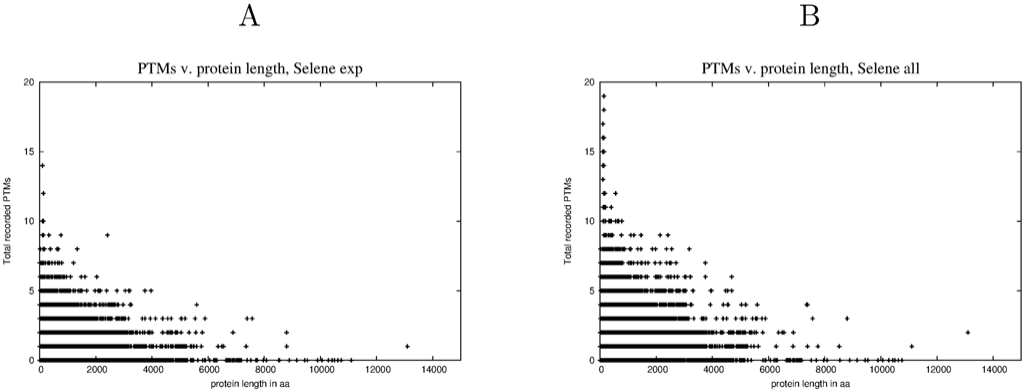
PTM distributions. A The distribution of PTMs against the length of a protein for *P*_*exp*_. B The distribution of PTMs against the length of a protein for *P*_*all*_.

The likely undercounting of PTM amino acids in the databases could pose a problem for this analysis if the full (and presently unknown) distribution of PTM was very different from that represented in *P*_*exp*_. While we cannot answer this question definitively, to gain a better perspective on the distribution of PTM the actual recorded PTMs for *P*_*exp*_ and *P*_*all*_ were compared (Fig 7A and 7B). These make clear that the additional but non-experimentally verified PTM in *P*_*all*_ [32], act in a self-similar way to those experimentally verified in *P*_*exp*_ in that no particular protein length appears to be favored by the inclusion of sites inferred but not verified experimentally. This self-similar behaviour of increasing discovery is very similar to the scale independent properties of the power-law behaviour shown in Fig 5A.

### Implications of this Study

The results presented here strongly suggest that the global properties (and therefore evolution) of the system of proteins investigated are constrained within structural bounds set by a conservation principle derived from information theory. The suggestion that such a conservation principle, regardless of natural selection, shapes the structural properties of the system of proteins is counter-intutive but nevertheless supported by the analyses presented here.
